# “It’s beautiful and it’s messy and it’s tragic”: exploring the role of compassion in the eating disorder recovery processes of 2S/LGBTQ + Canadians

**DOI:** 10.1186/s40337-024-00981-6

**Published:** 2024-02-07

**Authors:** Megan White, Andrew Thomas, Megan Aston, Phillip Joy

**Affiliations:** 1 Applied Human Nutrition, Mount Saint Vincent University, Halifax, NS Canada; 2 School of Nursing, Dalhousie University, Halifax, Canada

**Keywords:** Eating disorders, 2S/LGBTQ +, Gender minorities, Sexual minorities, Compassion, Poststructuralism, Foucauldian discourse analysis

## Abstract

This research explores experiences of compassion among 2S/LGBTQ + Canadians living with eating disorders in the context of eating disorder treatment and community support. There is a growing body of scholarship showing disparities in eating disorder care for those within 2S/LGBTQ + communities. Among the reported concerns is a potential lack of compassion in eating disorder treatment and recovery settings, something which may serve to exacerbate feelings of isolation and perpetuate misunderstandings of 2S/LGBTQ + people’s experiences. In an effort to understand these dynamics more deeply, we conducted semi-structured interviews with 2S/LGBTQ + Canadians who have experienced eating disorder care. The data collected were then subjected to Foucauldian discourse analysis, which produced three interconnected discursive considerations: feeling lack of structural compassion, 2S/LGBTQ + communities as places of respite, and 2S/LGBTQ + caregiving. One of the common threads among these discursive considerations was cis-heteronormativity ingrained in eating disorder treatment settings and health care systems more broadly. Our findings underscore the critical need for more enhanced compassion for 2S/LGBTQ + patients in eating disorder care settings. We conclude that compassion, when implemented on the levels of individual clinicians, policy and procedure, and institutions, may represent an avenue toward disrupting ingrained cis-heteronormativity and the associated discursive power structures contained in health care systems.

## Abstract (plain language summary)

Two-Spirit, lesbian, gay, bisexual, transgender, queer and other sexually or gender diverse (2S/LGBTQ+) are at increased risk for eating disorders often related to minority stress, discrimination, and heteronormative gender expectations. Compassion is believed to be a fundamental aspect of healthcare that builds human connections and enhances positive outcomes. Healthcare, however, is steeped in heteronormative assumptions that may further isolate many 2S/LGBTQ+ patients. This study explored how 2S/LGBTQ+ Canadians living with eating disorders felt about their care and the role of compassion in their recovery journeys. We found that these individuals often felt misunderstood and isolated during treatment. However, they often experienced understanding by connecting with other 2S/LGBTQ+ people both in care and in the community, which provided the compassionate spaces lacking in their healthcare experiences. These findings highlight a need for making healthcare more compassionate for 2S/LGBTQ+ people. This can be done by changing policies, mandatory training for healthcare professionals, and treatment that recognizes and discusses minority stresses, uses trauma-informed practices, and gender-affirming approaches. By doing such items, standard norms can be challenged and the care for 2S/LGBTQ+ people living with eating disorders can be improved.

## Introduction

Describing the meanings of compassion has been the purview of philosophers, theologians, and scholars for generations. Compassion can be considered as a feeling or an “orientation of the mind” [1], p. 145) that recognizes the suffering and pain of a person. Importantly, however, compassion in this view also involves an action to alleviate that suffering and pain [2]. We must also recognize that compassion is not merely an interpersonal attribute. Compassion is shaped by and contingent upon social norms discourses and knowledge, including those about gender and sexuality, which are in turn shaped by relations of power within society. This can affect who gets to give and receive compassion as well as both the delivery and reception of compassion [3]. Cannon [3] further notes that there is an existing social discourse that creates beliefs that not everyone is considered deserving of compassion. Society may deem certain sufferings as trivial or the individuals themselves as unworthy due to their actions or the nature of their suffering. For example, homophobic and transphobic ideologies often position Two-Spirit, lesbian, gay, bisexual, transgender, queer and other sexually or gender diverse (2S/LGBTQ +) people as unworthy of compassion. Cannon [3], however, emphasizes that compassion, like care and love, should not be abstracted from its particular context into a universal one.

Compassion is thought to be fundamental for positive human connections and a vital component in healthcare and therapeutic processes [4–8]. Historically, healthcare is home to relations of power which are the product of biomedical discourses, knowledge, and cis-heteronormative assumptions. Cis-heteronormative assumptions are societal assumptions that being cisgender and heterosexual are the norm. Such assumptions result in the surveillance of bodies creating pathologizing and marginalizing effects of those who fall outside of cis-heteronormativity [9–11], such as 2S/LGBTQ + people. Healthcare, including eating disorder care, is therefore imbued with discourses of cis-heteronormativity which position 2S/LGBTQ + bodies as inherently requiring discipline or correction. Previous research shows that 2S/LGBTQ + people face struggles in accessing healthcare and, when they do, they often encounter additional challenges in receiving safe and appropriate care [12, 13].

Eating disorders are multifaceted conditions influenced by psychological, sociocultural, and biological factors [14]. 2S/LGBTQ + communities are at heightened risk for eating disorders and often experience more problematic eating attitudes and behaviors. According to the Sex Now Survey, Canada’s largest and longest running survey of gay, bisexual, trans, Two-spirit, and queer (GBT2Q) men’s health, from 2018 to 2021, when asked about specific issues that participants wanted help with, approximately 35% reported wanting help with body image and 15% reported wanting help with an eating disorder [15].

Several reasons contribute to this heightened risk. Jones and Malson [16] report that not meeting dominant heteronormative gender expectations can contribute to the development of eating disorders for some lesbian women. Another reason for heightened eating disorder risk for 2S/LGBTQ + people is related to minority stress [17]. The minority stress model, developed by Meyer [18], refers to the chronically high levels of stress faced by members of stigmatized minority groups. It posits that minority stress is unique from the general stressors that all people face and can include stress from colonial violence, homophobia, biphobia, transphobia, prejudice, denial of civil and human rights, abuse, harassment, and family rejection. For example, how colonial structures, such as residential schools, were instrumental in installing settler perspectives of gender and sexuality on Indigenous people that continue to harm people today [19]. Additionally, for some 2S/LGBTQ + groups racism, cissexism, and misogyny often intersect and can lead to more severe eating disorder psychopathology compared to those with a single minority identity [20].

Furthermore, in eating disorder care remains interlinked with relations of power within society that reflect broader societal structures and cis-heteronormativity, which serve to marginalize 2S/LGBTQ + people during their care. For example, in a study of licensed therapists working in eating disorder treatment, cis-heteronormative structures were reported within the theme of clinical biases and microaggressions, as well as lack of training for cultural competence [21]. The work of Holmes [22] notes that the social aspects of eating disorders, especially those concerning gender, are often relegated to secondary factors in treatment contexts and, if considered, is mostly considered as colonial binary cis constructs of femininity. This can have negative consequences for care for those of diverse genders, including men, Two-Spirit, trans, nonbinary, gender queer, and agender people. Research has shown that 2S/LGBTQ + people living with eating disorders often feel that treatment is not created for them which can have negative impacts on recovery [23]. Another study that examined the treatment experiences of transgender individuals with eating disorders reported that out of the 84 participants, none of them had a positive experience during the treatment of their eating disorder [24]. A study with gender diverse Canadians found that existing eating disorder treatments, primarily designed for thin, white, cisgender women, fail to address the needs of gender diverse individuals, fostering feelings of isolation and neglecting the relationship between gender dysphoria and eating disorders [25]. Challenges include substantial access barriers, such as fears around being outed, and treatment strategies that do not account for gender dysphoria, compounded by a general lack of gender-affirming care from inadequately trained health professionals [25]. LaMarre et al. [26] suggests that both biological and sociocultural issues, including gender, must be give importance in eating disorder care.

The cultivation of compassion in eating disorder treatment and care may be particularly helpful for 2S/LGBTQ + groups. Compassion-focused therapy, initially developed to address feelings of self-criticism and shame, can be used in eating disorder treatment [27]. A central tenet of compassion-focused therapy emphasizes that mental health struggles are not personal failings but are the result of biology and the historical evolution of the human brain. Steindl et al. [27] also highlight that the use of compassion-focused therapy in eating disorder treatments can help patients recognize and understand the connection between their eating behaviours and emotions, nurture self-empathy, and boost self-confidence, self-awareness, assistance, and motivation. However, the minority stresses experienced from facing homophobia and transphobia are not biological or from any evolutionary processes but are trauma. Erikson [28] defines trauma as.

A blow to the tissues of the body—or, more frequently now, to the tissues of the mind—that results in an injury or some other disturbance. It is not an infection welling up from within, then. It is not a growth or a rupture or a blockage that originate inside. It is an assault from outside that breaks into the space once occupies as a person and damages the interior. Trauma, in this usage, refers not to the injury but to the blow that inflicted it, not to the state of mind but to the event that provoked it (p. 455).

In other words, the trauma many 2S/LGBTQ + experience is inflicted through encounters within day-to-day environments like schools, offices, religious spaces, and public areas as a result of with cis-heteronormative social discourses and knowledge. Such trauma has been compared to as “open wounds” that often makes self-compassion more difficult for 2S/LGBTQ + people [29]. Therefore, if compassion-focused therapy only focuses on the individual aspects of self-empathy, self-confidence, and self-awareness, without addressing the larger social structures like cis-heteronormativity that inflicts “open wounds” of trauma onto 2S/LGBTQ + individual then compassion-focused therapy may be more difficult. There have been calls to develop diagnostic tools, prevention efforts, and treatment programs tailored to the specific needs of 2S/LGBTQ + populations [20].

The current research, therefore, aims to explore the meanings of compassion in the therapeutic relationships of 2S/LGBTQ + Canadians living with eating disorders. To that end, we pose the research question: *What is the role of compassion in the eating disorder treatment processes for 2S/LGBTQ* + *Canadians and how do they understand compassion and its role in their treatment journey?* This research provides information which is both timely and clinically relevant for more 2S/LGBTQ + inclusive, compassionate, and effective eating disorder treatment and support programmes.

## Methodology & theoretical approach

A poststructural theoretical lens underpins this qualitative research. Poststructuralism is an approach which recognizes the socially constructed nature of identity and experience, and which challenges the notion of a singular universal truth. Further, it interrogates how people’s worldviews are formed through discourse, language, knowledge, and relations of power [30]. Poststructuralism can provide a framework for qualitative researchers because it seeks to challenge the way people experience the world by dissecting “regimes of truth” including those which inform the practices of healthcare institutions [30]. According to Michel Foucault [10], “regimes of truth” are the various systems within society that produce and maintain what is accepted as truth. These regimes are not about the truth in an absolute sense but rather the mechanisms and discourses that produce the norms of truth within a particular society. For Foucault, truth is not simply discovered but constructed through specific power relations and knowledge systems. These regimes are the rules by which we come to agree on what counts as true, governing what is acceptable to say, think, and do within a given society. For example, cis-heteronormativity, the assumption that being cis and straight is the “natural” way of being is accepted as a social truth because of the social discourses and the systems of power that make it so. Regimes of truth are tied to the institutions that control and disseminate knowledge, such as health institutions, and enforce and legitimize what is considered true, shaping societal norms, beliefs, and behaviors [10]. The  central salient concern for poststructuralist researchers is how knowledge becomes possible within various social and historical contexts [31]. To answer such a question, researchers must consider more than simple cause and effect mechanisms by examining the social contexts within which individuals live as well as how those contexts are constructed along axes such as class, race, gender, sexuality, culture, and history [31].

Our approach to this research was also informed by Richardson, who posits that ‘validation,’ in the context of qualitative poststructural research, should be considered a crystal rather than the more traditional triangle representation [32, 33]. The shape of a crystal more accurately symbolizes the multidimensionalities and myriad angles of approach to this kind of research. With the present study, our angles of approach included asking participants to review their interview transcripts, analyzing interview transcripts in the context of cis-heteronormativity, relations of power within care, and socially constructed nature of compassion, independently coding transcripts, and finalizing discursive considerations collaboratively.

Within poststructural research it is epistemologically important that researchers reflect upon their positionalities in relation to the topics they explore. The research team consists of a PhD Registered Dietitian, a PhD Registered Nurse, two graduate students, one who is a Registered Nurse and an eating disorder survivor. Three research team members identify as part of 2S/LGBTQ + communities, two cisgender and gay men, and a cisgender queer woman. All 2S/LGBTQ + team members have suffered homophobia growing up in small towns in Canada, informing their experiences of eating, health, and compassion. The other team member identifies as a cisgender straight woman and is seen as a strong 2S/LGBTQ + ally by the community. Three team members are of European descent, and one is of Indigenous descent. All members of the team are committed to furthering conversations about the needs of 2S/LGBTQ + people in eating disorder treatment programs, and the ways in which compassion can play a role in their recoveries.

## Methods

The inclusion criteria to participate in this study were to self-identify as sexually and/or gender diverse, be above the age of majority (19 years old), self-report having or having had eating disorders or disordered eating patterns, reside in Canada, and be able to speak and understand English. Our team developed text and image recruitment posts which were shared through (1) social media (Twitter, Instagram, and Facebook ads), (2) community organizations serving those with eating disorders and/or serving 2S/LGBTQ + Canadians, and (3) researchers’ networks. The research assistants’ email address was provided in recruitment postings as the point of contact for potential participants. Each potential participant was sent an informed consent document which contained the objectives of, and reasons for, the study and given the opportunity to ask questions or seek clarification. Once signed, the consent forms were returned to the research assistant and an interview was scheduled at the convenience of the participant.

Each participant completed a semi-structured virtual interview through Microsoft Teams™ (Microsoft, version 1.4.00.19572, Washington, 2017) from December 2022 to August 2023. Before the start of the questions, the consent form was reviewed by the interviewer with the participant to ensure continued informed consent. Interview questions were open-ended and designed with a poststructuralist framing to explore the lived experiences of 2S/LGBTQ + individuals. Demographic questions regarding gender identity, sexual orientation, age, location, ethnicity, and income were included and purposefully made open-ended to allow participants to self-identify in any way(s) that felt most appropriate for them. Interview questions focused on the intersection of their sexual and gender identities with their eating disorder and care. Additionally, the questions delved into the concept of compassion, its meaning for participants, and its particular significance for 2S/LGBTQ+ people. Questions relating to the expression of compassion during treatment and its role in their journey were asked. Interviews lasted approximately 75 min, were audio/video recorded, and transcribed in Microsoft Word (Microsoft Corporation). Each participant received a $25.00 (CAD) honorarium for their participation. The transcripts were then anonymized, and all participants were assigned a pseudonym.

The data were analyzed using Foucauldian discourse analysis, which is a process that involves systematic and critical examination of the data that looks beyond the surface meaning to situate the texts within historical, political, cultural, and social contexts (Cheek 1999). Foucauldian Discourse Analysis does not seek to uncover the literal meaning of what is said or unsaid, recognizing that discourses are sets of rules and procedures shaping how objects are understood and controlled [34]. In other words, discourses do not directly determine reality or singular truth but influence what can be known or practiced. This approach helps to provide insights into how individuals see themselves as subjects of knowledge. This process is often described as exploring 'focal points of experience' along three axes: knowledge (rules of discursive practices defining truth), power (rationalities governing others' conduct), and ethics (practices for self-constitution as a subject) that provides insights into the constructed nature of social reality [34].

Although there is no set process to follow when doing Foucauldian discourse analysis, the first step involved the researchers reviewing the interview transcripts independently multiple times. Each researcher made notes about the beliefs, values, and practices of the participants in relation to the research questions, keeping in mind discourses of cis-heteronormativity, relations of power within care, and socially constructed nature of compassion. Each researcher then produced preliminary codes that were grouped similarly. The second step consisted of the full research team coming together and reviewing collaboratively the notes, codes, and groupings from the independent analyses [34]. Through an iterative process, researchers then collectively finalized the groupings into discursive considerations constructed through our poststructural lens. This study received ethics approval from the Research Ethics Boards at Mount Saint Vincent University.

## Findings

Fourteen participants were recruited to the study. Participants’ self-reported demographic information can be found in Table 1. We present three interconnected discursive considerations: (*i*) feeling lack of structural compassion, (*ii*) 2S/LGBTQ + communities as places of respite, and (*iii*) 2S/LGBTQ + caregiving (Fig. 1).

**Table 1:** Participants’ self-reported demographic information

#	Pseudonym	Age	Sexuality	Ethnicity	Gender	Pronouns
1	Briar	27	Queer	White	Nonbinary trans	They/them
2	Kelly	29	Queer	White	Gender fluid	She/they
3	Calvin	23	Bisexual	Did not choose to disclose	Cisgender man	He/him
4	Karissa	22	Bisexual	African	Transgender woman	She/her
5	Daniel	42	Gay	Filipino	Cisgender man	He/him
6	Dina	26	Queer	Chose not to disclose	Cisgender woman	Chose not to disclose
7	Laurel	Chose not to disclose	Queer	Chose not to disclose	Cisgender woman	She/her
8	Patricia	35	Queer	Chose not to disclose	Cisgender woman	She/her
9	Analetta	24	Lesbian	French Canadian, Romani	Nonbinary	They/them
10	Kit	20	Queer	First generation immigrant	Does not identify with any gender	Any
11	Jodie	28	Pansexual	Indigenous	Nonbinary	She/they
12	Nicole	35	Pansexual	European Canadian	Cisgender woman	She/her
13	Mary	27	Lesbian	White	Cisgender woman	She/her
14	Jesse	Chose not to disclose	Queer	Chose not to disclose	Transmasculine nonbinary	They/he

**Fig. 1:**
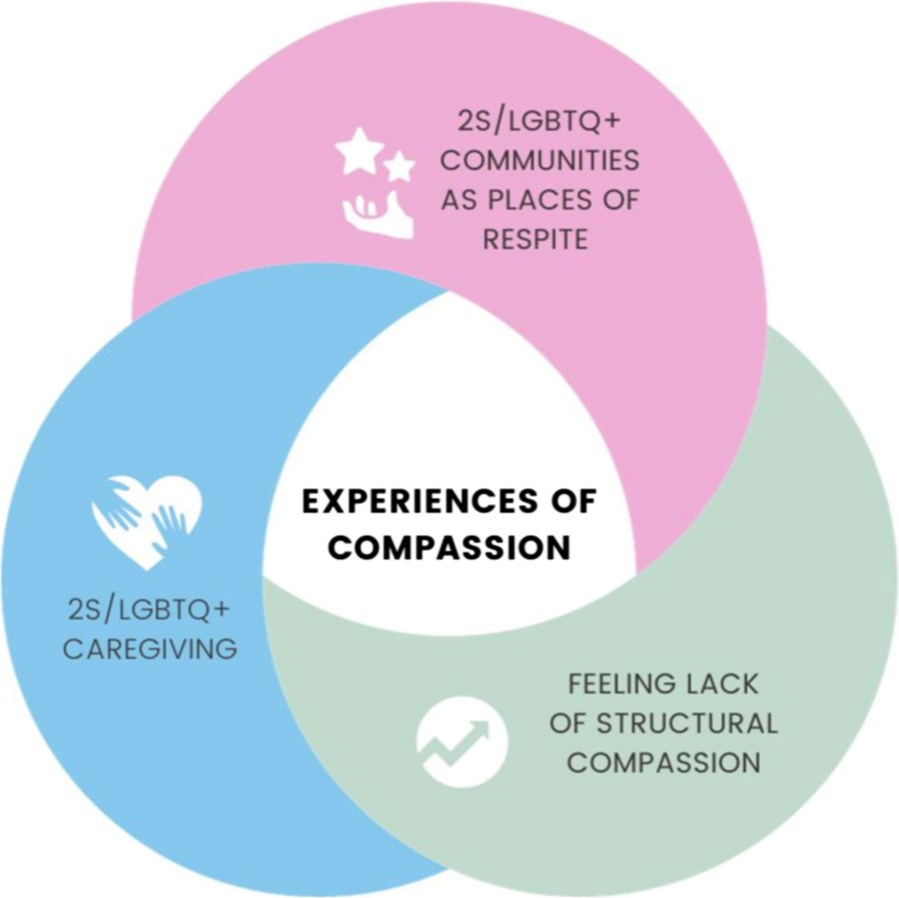
Interconnected discursive considerations of compassion

### Feeling lack of structural compassion

When we refer to ‘structural compassion,’ we mean something similar to the concepts of *structural competency* and *organizational compassion*. Structural competency refers to the trained ability to discern the ways in which people’s health statuses and related attitudes “represent the downstream implications of a number of upstream decisions about matters such as healthcare” [35], p. S140). That is, health and wellbeing occur within sociopolitical and economic contexts and are impacted by the actions of decision makers at the upper levels of social and institutional structures. Marginalized groups are disproportionately negatively impacted by these upstream decisions. Organizational compassion refers to the efforts of organizations and institutions to embed ethos of compassion within their policy objectives and operational procedures [36]. While participants recognized that individual healthcare practitioners have an important role to play in providing compassionate healthcare, they often contextualized clinicians’ ability to do so within larger systems and organizational structures. For example, Analetta, who identified as a nonbinary lesbian, commented, “I think a lot of people in positions of power that have the ability to make change lack understanding and compassion for queer people.” This comment shapes Annaletta’s knowledge that individuals who can enact transformational changes in healthcare often show an inability to show compassion, or a lack of knowledge of the need to show compassion, towards the experiences of 2S/LGBTQ + individuals.

When asked, almost every participant stated they felt the main barrier to structurally compassionate eating disorder treatment settings was the cis-heteronormativity ingrained in healthcare systems. Participants connected cis-heteronormativity to inadequately compassionate care and unilaterally described such attitudes and beliefs as fundamentally antithetical to compassion. Dina, a queer cisgender woman, expressed,

I feel like it (compassion in eating disorder recovery) is relevant in how heteronormative all of my eating disorder treatment was. When I was first diagnosed, there was a huge emphasis on, didn’t I ever wanna get pregnant? Didn’t I wanna have relationships (with men)? All of a sudden, *that* was the pathology. *That* was the proof of disorder.

In the quotation above, Dina described an experience which she characterized as uncompassionate. She felt that clinicians were appealing to her future to motivate her in recovery, but the examples they offered were heteronormative, making Dina feel as though her 2S/LGBTQ + identity was pathologized more than her eating disorder. Dina recognized that, at the point of care, she was positioned amid discourses of cis-heteronormativity in healthcare; she was made to feel that her romantic and sexual attractions, fertility, and, ultimately, her bodily autonomy as a 2S/LGBTQ + person were ‘othered’ and, as such, in need of correcting.

Dina further described her how exclusionary her eating disorder treatment and resources were for her, noting that.

I do think that the conventional inpatient treatment and in books—like, love your curves, love your body, let’s have role models for you that are all incredibly feminine and straight. And also, just very white, and young and middle class was so foreign that I couldn’t … I hated it. It wasn’t just that I couldn’t relate to it, but I felt like I so … Like, I couldn’t take people seriously. Like, you’re clearly not taking me seriously that I can’t stand my body and I’m uncomfortable with it, and you’re being like, ‘you’re beautiful!’ and like, that’s not even …. that’s not even in the same language.

Such cis-heteronormative discourse within treatment and resources can, as Dina described, be exclusionary and unrelatable for many 2S/LGBTQ + , especially those who are not white nor thin.

Mary, who identified as a cisgender lesbian, offered another example of discourses of cis-heteronormativity in healthcare settings,

It’s about understanding that we live in a heteronormative society. When you’re queer, you’re the outlier. You’re not the standard, the norm. I think to be compassionate would be to reframe your language. For example, when I access healthcare, a lot of times people assume I have a male partner. And that’s really hurtful for me. I don’t usually make a big stink out of it because I’m just kind of used to it.

Like many of the other participants, Mary’s knowledge about herself and the care that she received in her eating disorder treatment settings was informed by discourses of cis-heteronormativity ingrained in healthcare systems. She believed disrupting discourses of cis-heteronormativity through practices of reframing language within care would be helpful.

Many participants shared that they often found themselves strategically concealing or modifying their gender expression and sexuality, or controlling the extent of information about these aspects they disclosed to their healthcare providers. Further, they felt those decisions were dependent upon the context, environment, and their in-the-moment assessment of the attitudes of the people with whom they were speaking. Dina, when asked how she defines her gender, stated,

When I’m acknowledging privilege, I’ll say that I’m officially cis. You have to look at what community [you’re among] and decide, yeah, it’s safer here to say that ‘I’m relatively cis.’

Dina made her decisions about how much of her gender identity to express based on her knowledge of the relative safety of the situation she was in. When asked about their experiences with altering or concealing their gender identities and/or sexualities, participants consistently reported they often assessed eating disorder treatment settings as being unsafe. Several participants elaborated, stating that they felt the primary reason for believing treatment settings were unsafe were the cis- binary gender normative practices they experienced within these settings. Dina further commented that.

…Entering eating disorder treatment was one of the strongest times when I was aware of gender norms because it’s such a gendered environment. I became aware that, as a woman, I should have a body image when I didn’t before. I was just a kid. Suddenly, there were questions about my body as a female body that did not ring as relevant to me. And then just how gendered both healthcare and eating disorder care is, constantly being called a girl and having passive femininity demanded of us all the time. It seemed so intrinsic to it being a feminine diagnosis.

For Dina, the gendered nature on the hospital environment and eating disorder care created knowledge about her body that she previously did not have. Similarly, Jodie, who identified as pansexual and nonbinary, described another experience of gender assumptions in eating disorder care,

I feel like they [psychologists] were like, well, if the bulk of what’s happened to you happened when you were 16, then that’s just the teenage girl experience.

The gender normativity Dina and Jodie experienced in eating disorder recovery settings was based in discourses of cis-heteronormativity, which made those places unsafe for expressing their genders and sexualities authentically.

Several participants offered suggestions for improving 2S/LGBTQ + structural compassion in eating disorder recovery care. Among these suggestions were targeted 2S/LGBTQ + training and education for care providers, and trauma-informed approaches that acknowledge the harm and trauma suffered by 2S/LGBTQ + people as a result of societal cis-heteronormativity, colonialism, racism, homophobia, and transphobia to procedures and policy. Briar, who identified as queer, nonbinary trans, commented on staff training with respect to compassion, stating,

Hire more people who are competent in dealing with eating disorders from a lens that incorporates gender and sexuality beyond the cis, straight, white, skinny female. We need to get beyond that, and I think that until you start staffing people who are competent in those areas, you’re setting it up for failure. In terms of the hiring process, the training process, people need to be more informed because otherwise you’re just forcing queer people to provide that education for you, and that’s not their job. They’re there to heal, not to educate, and unfortunately, that’s what a lot of queer people end up doing; educating people.

For Briar, having 2S/LGBTQ + healthcare workers and training for all care providers was essential to disrupting cis-heteronormativity within eating disorder treatment.

Another participant, Kelly, who identified as queer and gender fluid, described how trauma-informed approaches helped her experience compassion in her eating disorder recovery.

I know lots of people who had terrible experiences in treatment. I happened to have an amazing experience in treatment. The doctors and the clinicians there had a lot of compassion for me. I think that they were able to clearly see how sick I was in a way that I couldn’t. They also understood that I had a lot of other stuff happening. I had some pretty severe trauma and attachment shit going on.

Kelly constructed her experiences in treatment as positive because her doctors and the clinicians were able to recognize the severe trauma that being queer in a cis-heteronormative world can inflict on people.

Kelly further went on to note that, in order to be compassionate in their care for her, clinicians sometimes had to ‘bend’ the rules, indicating that she situated individual practitioners’ compassion within larger structural and organizational contexts.

They absolutely bent the rules for me. At the time the eating disorder program was about eight months long, and I know that it’s even significantly shorter now. But they let me stay in that program for a year and a half. She [Kelly’s psychologist] told me that the hospital higher-ups were looking at her records and telling her she’d been seeing me longer than she should have. She had to push back and say, ‘my client and I decide that.’ I’m still reaping the benefits of that compassion and that willingness to be flexible and to meet me where I am and help me access things.

Through this quote, Kelly’s knowledge of compassionate care is constructed through relations of power within healthcare structures, in which clinicians sometimes must juggle their ability to provide compassionate care with organizational policies and regulations, especially for 2S/LGBTQ + people who face minority stress.

### 2S/LGBTQ + communities as places of respite

Some participants drew a clear connection between their experiences with cis-heteronormativity in eating disorder care and feelings of exhaustion. These participants stated they felt this exhaustion was a direct result of feeling the need to strategically alter or hide their gender presentation and/or their sexuality as discussed in the first discursive consideration. When asked about this exhaustion, Briar told the interviewer,

It [hiding or altering gender presentation] is emotionally exhausting. But the alternative is also exhausting, so there’s really no escaping what that looks like. There is no escaping the exhaustion. It’s just choosing what you’re gonna put up with.

In the statement above, Briar described feeling that they must contend with one form or another of exhaustion. Such feelings of exhaustion are created from discourses of cis-heteronormativity in healthcare that positions 2S/LGBTQ + people as needing to justify their existence outside of social norms. Interestingly, they use the phrase ‘no escape,’ suggesting that they viewed this exhaustion as an inevitable outcome for 2S/LGBTQ + people living within a cis-heteronormative world, particularly in healthcare systems in which they subjugated to binary expectations of gender, bodies, and behaviors.

When asked about 2S/LGBTQ + communities and how they think compassion manifests within them, participants overwhelmingly described those communities as places of respite, safety, and shared experiential understanding. Participants consistently characterized queer communities as places in which they were not required to hide or to explain themselves; they can simply exist, and their gender and sexual identities are respected. Nicole, who identified as a pansexual cisgender woman, explained,

There’s a level of safety in those groups [queer communities]. There’s openness and freedom to change and grow. Especially in my roller derby group. People can change and grow and be accepted and celebrated. There’s this thing we do, we have a check-in, we sit in a circle, and everyone talks about what’s going on with them. There’s a space that’s held there that’s really beautiful. The openness, inclusivity, and compassion that we carry for each other’s differences, and celebrating those differences.

Nicole’s experiences within her queer community group were constructed through discourses of shared experience among 2S/LGBTQ + people that she saw as compassionate. Compassion, for Nicole, were acts of safety, checking-in with others, and celebrating differences. Daniel, who identified as a gay cisgender man, echoed Nicole’s sentiment, saying, “I think compassion in the LGBT + community is, first and foremost, about acceptance. I think compassion is the acceptance and celebration of difference.”

Kelly offered a similar sentiment, explaining to the interviewer,

The way that I see compassion showing up in queer communities is the acceptance of difference. I think that rigid standards of conformity are about as far from compassion as you can get. The diversity that exists within the queer community is a pretty clear expression of compassion. When I’m in a room full of people where I don’t know anybody, I can usually find the other queer person in the room and be with them because there’s this level of comfort. I have the feeling that I can already trust you in some ways that I may not be able to trust other people, and I can let my guard down.

For Kelly, having shared experiences of living in a cis-heteronormative world that has rigid standards of conformity about gender and sexuality created feelings of safety and trust with other 2S/LGBTQ + people. Kelly positioned this discourse of shared experience as fertile soil for compassion. Jesse, who identified as transmasculine and nonbinary, expanded on the discourse of shared experiential understanding in queer communities and how those shared experiences manifest as compassion, telling the interviewer,

There’s always that unspoken thing that brings queer people together. Inherently, at some point in your life, you’re going to have had someone not be accepting of your identity or not be accepting of you in some way, shape, or form. And though this is common for any community, cis-heteronormative or not, it’s different for us because it’s specifically our gender, our identity, specifically our sexuality. It’s that fundamental thing that we can’t change about ourselves, and that people focus on that really shouldn’t affect or matter to anyone else. And so, because we all have that shared experience, we have a little bit of commonality that makes us a community.

Nearly all participants situated 2S/LGBTQ + communities as places in which they could arrive and feel safe, understood, and in which they could avoid the emotional exhaustion of explaining, altering, or hiding their identities. This was contrasted with their experiences within highly gender- and cis-heteronormative eating disorder recovery settings. Participants noted the need to continuously assess the safety of recovery settings and personnel, alter their presentation and explain their identities. As previously stated, such needs were noted to result in feelings of exhaustion for the participants, which detracted from their treatment processes as well as their overall wellbeing.

### 2S/LGBTQ + Caregiving

The majority of our participants spoke at some length about 2S/LGBTQ + people caring for one another during their eating disorder journey, as well as while receiving gender-affirming care, something which they saw as intimately related to their body image and disordered eating patterns. Briar recalled asking a care provider in a treatment setting, “Do you understand that part of my restrictive eating is because I don’t feel affirmed in my gender? That’s how I’m going to manipulate my body to make myself feel affirmed.” Briar, when asked how they were able to navigate eating disorder recovery despite their negative experiences within treatment settings, described finding the most helpful supports within 2S/LGBTQ + communities, stating,

What ends up happening is that you create a community, and you create an environment of support amongst yourselves because you have no other option. It’s beautiful and it’s messy and it’s tragic that we even have to be in that position to begin with. It's unfortunate that we can’t just access the services that we need and that we're forced to be those emotional supports to other people whenever we're probably so low on our own capacity, but we're wanting to support our own, and so we make those sacrifices because of that. Yeah, it’s just something that you see in the queer community because it’s necessary. Otherwise, none of us would make it out the other end, you know?

This quotation encapsulates a sentiment several participants expressed; when healthcare settings are uncompassionate or structurally incompetent, 2S/LGBTQ + people turn to one another for the compassionate care that they feel should have been delivered by providers. That said, many participants expressed conflicting feelings about this. On one hand, they expressed disappointment that eating disorder treatment and broader healthcare settings often do not meet their needs but, on the other hand, they expressed gratitude for the intracommunity compassionate care they received from fellow 2S/LGBTQ + people. Jesse shared,

The [2S/LGBTQ+] community’s just so wanting to share resources. I’ve seen it all over social media, all kinds of different platforms where people are trying to explain and share every aspect of what [gender-affirming] surgery might look like. This is what the recovery time looks like. This is what your chest might look like. You might have these drains. And that’s how people are learning these things. They’re not learning them from their healthcare providers. They’re doing their own research and sharing it with the community. That is truly compassion, in my eyes.

Dina offered another example of 2S/LGBTQ + people providing care for one another,

I have a friend who is receiving palliative care for her eating disorder, and I’ve been visiting her in hospital daily, and so I feel like recently, my relations with this [eating disorder care] has been one of advocacy and community caregiving with individual people.

Interestingly, participants also described this kind of intracommunity caretaking happening within eating disorder treatment, including inpatient hospital settings. Kelly recalled such an experience, stating,

After we ate breakfast and the clinicians had left the kitchen, I looked at one of my co-patients there and I was like, so, do we actually follow these rules? Like, if I did wanna purge where would I go? Like, where is safe? And I still consider this to be one of the most generous experiences of compassion I've had. She just looked at me. She had been there a while and she was like, ‘how about instead of answering the question for you, why don't we go paint? There’re art supplies right here. How about we just sit down, and paint and we think about that later.’ And that's how I sat through my first 30 minutes after a meal at clinic. I had initially been very against the idea of a group therapy aspect. I really didn't think it was gonna be a good fit and I don't know that I would have stuck with it or that it would have been anywhere near as effective without my co-patients.

This discursive consideration interlocks with the second discursive consideration as participants saw queer communities not only as places for rest and acceptance, but also as safe places in which to access compassionate support in their eating disorder recovery from people who recognize their queerness as an important factor in that recovery process.

## Discussion

Eating disorders are complex psychiatric illnesses influenced by biological, psychological, and sociocultural factors. Treatment and care are multifaceted processes often requiring an array of therapeutic interventions. But these processes are also situated within social institutions and relations of power. As Lester [37] states, “care is a slippery term…the concept of care is deceptively banal, evoking a feel-good sense of tending to another’s needs, or an affective orientation of selfless compassion and empathy. But ‘‘care’’ is both more complex (and darker) than our everyday associations suggest. Whatever else it may be about, care is, at heart, about power” (p. 517). Lester’s work [37] highlights how cultural, social, and institutional dynamics shape the experiences and treatments of eating disorders and underscores the need for eating disorder treatment settings to be reconfigured as spaces where safety, affirmation of identities, and compassion are woven into the fabric of care. Such reconfigured environments would, therefore, transcend traditional boundaries and integrate an approach that is appropriate for 2S/LGBTQ + individuals. These environments may help 2S/LGBTQ + patients to feel safer with regard to disclosure of their gender and sexualities. Withholding disclosure of crucial elements of one's identity, particularly elements that are deeply intertwined with, and often contributes to, their eating disorder can hinder the development of trust, reciprocal respect, and the formation of a therapeutic relationship, which are all essential for effectively treating eating disorders [24].

Increasingly, compassion-focused therapy has been suggested as a beneficial approach for individuals with eating disorders [38]. Compassion in therapy is thought to be a skill that can be learned with the ultimate goal being the cultivation of a self-compassionate inner dialogue for clients, supplanting self-criticism and self-blame [39]. Compassion-focused therapy encourages individuals to develop and work with experiences of inner warmth, safeness, and self-soothing, countering feelings of shame, self-criticism, and internal hostility common for clients struggling with eating disorders [40]. Engaging in self-compassion exercises such as loving-kindness meditation has also shown promise in enhancing positive emotions and reducing negative ones in individuals [41]. Compassionate practices may enable patients to better navigate the challenges of recovery but, for some 2S/LGBTQ + people, being compassionate to oneself may be harder than for others. We have previously reported on how, for many 2S/LGBTQ + people, self-compassion can be difficult because of the ‘open wounds’ of trauma they have as a result of a violence, discrimination, homophobia, and transphobia of a cis-heteronormative society [29].

For 2S/LGBTQ + individuals living with eating disorders, their experiences are often compounded by fear of rejection, judgement, and socially perpetuated norms and standards around beauty, gender, and body image [42]. These feelings can be yet further exacerbated by instances of body dysphoria, especially among transgender people [23, 25, 29]. In discursive consideration one (feeling lack of structural compassion), participants described how they believe their treatments were negatively impacted by the lack of compassion within healthcare institutions. Indeed, literature on organizational compassion has recognized that healthcare practitioners’ efforts toward providing compassionate care frequently “conflict with organisational logics and policies” [36], p. 200), and that healthcare structures may function as an “array of impersonal organisations, institutions, and forms of discursive power” [43], p. 505). That is, the traditional power relations of medicine which are built into the structure and function of healthcare institutions pose barriers to compassionate care on the levels of individual clinicians, policies and procedures, and institutional culture(s).

Perhaps more importantly for our participants, though, were their experiences of cis-heteronormative discourses—which uphold heterosexual relationships and traditional gender roles as the norm—in their recovery journeys. Historically, narratives around treatment modalities for eating disorders have been predominantly centred on the experiences of straight, cisgender women and girls, and often neglected the unique experiences and challenges faced by 2S/LGBTQ + people [44]. Discourses of cis-heteronormativity in eating disorder treatment settings may serve to exacerbate feelings of isolation and invisibility among 2S/LGBTQ + patients as they may struggle to find their experiences reflected in the therapeutic process, as participants noted in this study, or inappropriate and harmful treatment interventions, as described by [25]. For a comprehensive and compassionate recovery process, participants believed it imperative that professionals acknowledge and actively challenge discourses of cis-heteronormativity without putting the burden on 2S/LGBTQ + patients to teach about ‘2S/LGBTQ + issues’. The participants saw this as part of compassionate therapy. Therefore, incorporating compassion into treatment and care processes involves a deliberate and active understanding suffering, minority stresses, and trauma faced by 2S/LGBTQ + patients. It also requires an engagement and a commitment from healthcare providers to cultivate an environment where 2S/LGBTQ + individuals receive care that is as emotionally supportive as it is physically safe.

Cis-heteronormative assumptions described in discursive consideration one (feeling lack of structural compassion) are deeply connected and interrelated to discursive consideration two (2S/LGBTQ + communities as places of respite) and discursive consideration three (2S/LGBTQ + caregiving) as participants saw discourses of cis-heteronormativity as the reason they need 2S/LGBTQ + communities of respite and 2S/LGBTQ + caregiving. Personal connections among members of the 2S/LGBTQ + community play a pivotal role in enhancing the mental, emotional, and social well-being of individuals [18, 45–48]. Such connections offer a sense of belonging and understanding, which can be profoundly meaningful, especially for those who may experience feelings of isolation or stigmatization.

Participants identified a discourse of shared experience in queer communities and believed that connections with other 2S/LGBTQ + people living with eating disorders was a form of compassion that helped their recovery journeys and acted as a kind of protective armor for navigating cis-heteronormative health care systems and accompanying discursive power structures. Other researchers have noted that by interacting with peers who share similar experiences and identities, 2S/LGBTQ + individuals often find supportive spaces where they can express themselves authentically, share their challenges, and seek guidance [49–51]. Participants saw their relationships with other 2S/LGBTQ + people as a safe harbour from the constant cis-heteronormativity within care practices that further isolated them during recovery and challenged their ability to move forward on their journey of health and wellbeing.

In healthcare settings, having 2S/LGBTQ + healthcare professionals can lead to more culturally safe, effective, and compassionate care. 2S/LGBTQ + people often report apprehension about seeking medical care due to fears of discrimination, misunderstanding, or insensitivity [52]. Participants believed that 2S/LGBTQ + healthcare professionals shared an understanding of many of the unique health and wellness concerns they faced, creating a more comfortable, trusting, effective, and compassionate relationship. The presence of more 2S/LGBTQ + healthcare professionals within clinical environments is not just beneficial—it is essential. Their representation can serve as a cornerstone for nurturing compassionate care that resonates with the lived experiences of 2S/LGBTQ + patients. By integrating 2S/LGBTQ + practitioners into healthcare teams, we can directly address the lack of compassion that many participants described. Moreover, the inclusion of 2S/LGBTQ + health professionals disrupts the cis-heteronormative institutions that currently exist and acts as a catalyst for change, ensuring that the care delivered is not only compassionate but responsive to the needs of 2S/LGBTQ + patients. This would be profound transformation in the delivery of eating disorder healthcare that can dismantle barriers and redefine the experiences of 2S/LGBTQ + patients. Additionally, perhaps having more 2S/LGBTQ + healthcare professionals within eating disorder care could also alleviate obstacles stemming from healthcare providers’ insufficient understanding or biases concerning 2S/LGBTQ + in treatment that other researchers [53] have noted.

Given the complexities of cis-heteronormative structures within healthcare, a compassion-focused approach becomes even more crucial. Compassion within eating disorder therapy for 2S/LGBTQ + people involves recognizing the many layers of stigmatization they may endure, such as those associated with eating disorders, stigmas around 2S/LGBTQ + identity, and, for some, stigma resulting from colonialization and racism [18, 54]. By integrating compassion into treatment strategies, 2S/LGBTQ + people living with eating disorders can become better equipped to address feelings of shame, self-criticism, and isolation [55], feelings that for many 2S/LGBTQ + people are the result of societal constructs of gender and sexual orientation. Furthermore, it has been noted that integrating affirmative, particularly gender-affirming, approaches that recognize and celebrate diverse genders, including trans and nonbinary, are critical for effective eating disorder treatments and the recovery journey [56–58].

While this study has explored compassion within eating disorder treatment and recovery for 2S/LGBTQ + people we acknowledge the breadth and depth of diversity within 2S/LGBTQ + communities. Each group with the rainbow umbrella encompasses a wide range of experiences and identities. Our analysis group these communities together but exploring each group with a dedicated focus could yield even more specific insights. Additionally, the intersections of colonialism and racism with 2S/LGBTQ + identities within eating disorder treatment presents a rich field for further research. The current study incorporates an understanding of these factors, but there is substantial potential for future research to delve deeper into the nuanced intersections of identities. The scope of this study was purposely designed to provide a broad understanding, laying the groundwork for more detailed investigations into these critical facets of eating disorder treatment. We encourage subsequent studies to build upon this foundation with targeted research that can inform structurally competent, culturally safe, and compassionate treatment modalities for 2S/LGBTQ + patients.

## Conclusion

It is imperative that treatment and care practices for eating disorders prioritize the disruption of structural oppressions for 2S/LGBTQ+ people because of cis-heteronormative discourses and knowledge. The question becomes how can such disruption occur? Participants gave some suggestions, such as hiring 2S/LGBTQ + healthcare professionals, providing 2S/LGBTQ + training opportunities to staff, and fostering connections with other 2S/LGBTQ + individuals during care. Eating disorder treatment centers could also critically evaluate their own policies and practices to identify how they may perpetuate cis-heteronormativity and negatively impact 2S/LGBTQ + patients. Such structural changes may lead to more compassionate care. The value of compassion through the disruption of cis-heteronormativity in eating disorder recovery cannot be overstated for 2S/LGBTQ + patients. Compassionate care, which recognizes and discusses minority stresses, uses trauma-informed and gender-affirming approaches, and fosters open communication is essential for effective treatment plans 2S/LGBTQ + individuals.

## Data Availability

Not applicable.
